# Supplementary material for: Common seed dispersers contribute most to the persistence of a fleshy-fruited tree
Source: Commun Biol. 2023 Mar 27;6:330. doi: 10.1038/s42003-023-04647-y (PMC10043030; doi:10.1038/s42003-023-04647-y)
Supplement: Supplementary file 2 — Supplementary Materials [file 42003_2023_4647_MOESM2_ESM.docx]

**Supplementary Information for**

**Common seed dispersers contribute most to the persistence of a fleshy-fruited tree**

**Authors**

Finn Rehling*^1,2^, Eelke Jongejans^3,4^, Jan Schlautmann^1^, Jörg Albrecht^5^, Hubert Fassbender^1^, Bogdan Jaroszewicz^6^, Diethart Matthies^7^, Lina Waldschmidt^1^, Nina Farwig^1^, Dana G. Schabo^1^

**Affiliations**

^1^University of Marburg, Department of Biology, Conservation Ecology, Marburg, Germany.

^2^University of Marburg, Department of Biology, Animal Ecology, Marburg, Germany.

^3^Radboud University, RIBES, Nijmegen, Netherlands.

^4^NIOO-KNAW, Department of Animal Ecology, Wageningen, Netherlands.

^5^Senckenberg Biodiversity and Climate Research Centre Frankfurt, Frankfurt, Germany.

^6^University of Warsaw, Faculty of Biology, Białowieża Geobotanical Station, Białowieża, Poland.

^7^University of Marburg, Department of Biology, Plant Ecology, Marburg, Germany.

*Corresponding author: Finn Rehling.

Email: [finn.rehling@nature.uni-freiburg.de](mailto:finn.rehling@nature.uni-freiburg.de)

**This file includes:**

Supplementary Figures 1-15

Supplementary Tables 1-5

Supplementary Discussions 1-3

Supplementary Methods 1

Supplementary References

Supplementary Table 1: Geographical coordinates and characteristics of the experimental setup in Białowieża Forest, Eastern Poland.

|  |  |  |  |  | Entire fleshy-fruited plant community | | | | Only *Frangula* | | | |
| --- | --- | --- | --- | --- | --- | --- | --- | --- | --- | --- | --- | --- |
| Plot | Site | Latitude | Longitude | Forest | Removal | Deposition | Demography | Recruitment | Removal | Deposition | Demography | Recruitment |
| 301 | 1 | 52.7425223 | 23.8331253 | Old-growth | 1 | 1 | 1 | 0 | 0 | 1 | 1 | 0 |
| 312 | 2 | 52.7894196 | 23.8446378 | Old-growth | 1 | 0 | 1 | 0 | 1 | 0 | 1 | 0 |
| 303 | 3 | 52.7793992 | 23.8580859 | Old-growth | 1 | 1 | 1 | 0 | 1 | 1 | 1 | 0 |
| 314 | 4 | 52.7306426 | 23.8221458 | Old-growth | 1 | 1 | 1 | 1 | 1 | 1 | 1 | 1 |
| 315 | 5 | 52.7987112 | 23.8260231 | Old-growth | 1 | 1 | 1 | 1 | 0 | 1 | 1 | 1 |
| 102 | 6 | 52.7030703 | 23.6535284 | Degraded | 1 | 1 | 1 | 1 | 1 | 1 | 1 | 1 |
| 103 | 7 | 52.6701456 | 23.6853848 | Degraded | 1 | 0 | 0 | 0 | 1 | 0 | 0 | 0 |
| 203 | 8 | 52.7042936 | 23.6224021 | Degraded | 1 | 1 | 1 | 0 | 1 | 1 | 1 | 0 |
| 111 | 9 | 52.7172336 | 23.8167656 | Degraded | 1 | 0 | 0 | 0 | 1 | 0 | 0 | 0 |
| 112 | 10 | 52.7338132 | 23.7892289 | Degraded | 1 | 1 | 1 | 0 | 1 | 1 | 1 | 0 |
| 30 | 11 | 52.6886727 | 23.8777491 | Degraded | 1 | 1 | 1 | 1 | 1 | 1 | 1 | 1 |
| 11 | 12 | 52.8552399 | 23.8399927 | Degraded | 1 | 0 | 1 | 0 | 1 | 0 | 1 | 0 |
| 36 | 13 | 52.8540699 | 23.7298022 | Degraded | 1 | 1 | 1 | 0 | 1 | 1 | 1 | 0 |
| 33 | 14 | 52.8453121 | 23.8097584 | Degraded | 1 | 0 | 0 | 0 | 1 | 0 | 0 | 0 |
| 13 | 15 | 52.8322092 | 23.7721042 | Degraded | 1 | 1 | 1 | 0 | 1 | 1 | 1 | 0 |
| 15 | 16 | 52.6917217 | 23.8407402 | Degraded | 1 | 1 | 1 | 0 | 1 | 1 | 1 | 0 |
| 35 | 17 | 52.8727648 | 23.7224447 | Degraded | 1 | 1 | 1 | 0 | 1 | 1 | 1 | 0 |

Supplementary Table 2: Summary of the sampling of seed removal, deposition and plant demography on *Frangula alnus* across 17 study sites in Białowieża Forest. The entries in each cell depict the year- and plot-specific sampling of (1) the number of individuals of *F. alnus* that have been selected for the three sessions of frugivore observations; (2) the number of scats with only *F. alnus* or all scats with seeds of the fleshy-fruited plant community found along the transects; (3) the number of individuals that were used for vital rate assessment of plant demography.

|  |  |  | **Seed removal** | |  | **Seed deposition** | | | | | | |  | **Plant demography** | | |
| --- | --- | --- | --- | --- | --- | --- | --- | --- | --- | --- | --- | --- | --- | --- | --- | --- |
|  |  |  |  |  |  | **Scats with seeds of *F. alnus*** | | |  | **All scats with seeds** | | |  |  |  |  |
| **Plot** | **Site** |  | **2011** | **2012** |  | **2016** | **2017** | **2018** |  | **2016** | **2017** | **2018** |  | **2017** | **2018** | **2019** |
| 301 | 1 |  |  |  |  |  |  |  |  | 5 | 6 | 119 |  |  |  |  |
| 303 | 3 |  | 1 |  |  | 0 | 0 | 19 |  | 17 | 18 | 141 |  | 12 | 7 | 6 |
| 312 | 2 |  | 3 |  |  |  |  |  |  |  |  |  |  | 98 | 80 | 55 |
| 314 | 4 |  | 3 | 3 |  | 2 | 7 | 17 |  | 4 | 8 | 29 |  | 82 | 43 | 34 |
| 315 | 5 |  | 1 |  |  | 1 | 0 | 1 |  | 15 | 9 | 82 |  | 13 | 14 | 16 |
| 102 | 6 |  | 2 | 2 |  | 0 | 0 | 0 |  | 1 | 1 | 3 |  | 0 | 2 | 4 |
| 103 | 7 |  | 3 |  |  |  |  |  |  |  |  |  |  |  |  |  |
| 203 | 8 |  | 3 | 3 |  | 0 | 0 | 35 |  | 22 | 6 | 63 |  | 5 | 5 | 4 |
| 111 | 9 |  | 2 | 2 |  |  |  |  |  |  |  |  |  |  |  |  |
| 112 | 10 |  | 3 | 3 |  | 6 | 62 | 68 |  | 29 | 115 | 111 |  | 14 | 20 | 32 |
| 30 | 11 |  |  | 3 |  | 11 | 8 | 49 |  | 22 | 28 | 138 |  | 53 | 30 | 55 |
| 11 | 12 |  |  | 3 |  |  |  |  |  |  |  |  |  | 25 | 26 | 17 |
| 36 | 13 |  |  | 2 |  | 0 | 1 | 4 |  | 24 | 31 | 85 |  | 19 | 15 | 6 |
| 33 | 14 |  |  | 2 |  |  |  |  |  |  |  |  |  |  |  |  |
| 13 | 15 |  |  | 3 |  | 13 | 25 | 21 |  | 42* | 67* | 120* |  | 18 | 19 | 19 |
| 15 | 16 |  |  | 2 |  | 2 | 1 | 0 |  | 35 | 59 | 49 |  | 11 | 8 | 16 |
| 35 | 17 |  |  | 3 |  | 3 | 5 | 14 |  | 33 | 8 | 184 |  | 15 | 16 | 24 |
| Total |  |  | 21 | 31 |  | 38 | 109 | 228 |  | 249 | 356 | 1124 |  | 365 | 285 | 288 |
| *We found almost 500 scats beneath one tree of *Sambucus nigra*, which acted as a hub tree in the local forest. We excluded scats from this transect other than *F. alnus* from the analyses, as these influenced the deposition pattern of frugivore species. | | | | | | | | | | | | | | | | |

Supplementary Table 3: List of codes, removal and deposition of animal seed disperser of *Frangula alnus* in Białowieża Forest.

| **Order** | | **Family** |  | **Animal species** | **Code** | **Visits** | **Prob. of handling fruit** | **Mean no. of handled fruit** | **Interaction frequency** | **No. of observed handling** | | | | **No. of scats with only *F. alnus*** | **No. of scats with all seeds*** |
| --- | --- | --- | --- | --- | --- | --- | --- | --- | --- | --- | --- | --- | --- | --- | --- |
|  | |  |  |  |  |  |  |  |  | **swallow** | **remove** | **crush** | **drop** |  |  |
| Passeriformes | | Fringillidae |  | *Coccothraustes coccothraustes* | 2 | 4 | 0.75 | 1.67 | 5.01 | 0 | 1 | 4 | 0 | 0 | 0 |
| Piciformes | | Picidae |  | *Dendrocopos major* | 3 | 1 | 1.00 | 1.00 | 1.00 | 11 | 0 | 0 | 0 | 0 | 1 |
| Piciformes | | Picidae |  | *Dendrocopos medius* | 4 | 7 | 0.86 | 1.83 | 11.02 | 1 | 0 | 0 | 0 | 0 | 0 |
| Passeriformes | | Muscicapidae |  | *Erithacus rubecula* | 6 | 135 | 0.73 | 1.89 | 185.97 | 155 | 5 | 0 | 1 | 7 | 30 |
| Passeriformes | | Muscicapidae |  | *Luscinia luscinia* | 7 | 4 | 1.00 | 1.50 | 6.00 | 6 | 0 | 0 | 0 | 0 | 3 |
| Passeriformes | | Muscicapidae |  | *Muscicapa striata* | 10 | 21 | 0.64 | 1.67 | 22.44 | 14 | 0 | 0 | 1 | 0 | 0 |
| Passeriformes | | Paridae |  | *Parus major* | 12 | 29 | 0.17 | 1.00 | 4.93 | 2 | 0 | 0 | 0 | 1 | 2 |
| Passeriformes | | Sittidae |  | *Sitta europaea* | 14 | 59 | 0.51 | 1.75 | 52.66 | 25 | 10 | 0 | 0 | 0 | 0 |
| Passeriformes | | Sylviidae |  | *Sylvia atricapilla* | 15 | 582 | 0.90 | 2.49 | 1304.26 | 935 | 5 | 0 | 5 | 232 | 570 |
| Passeriformes | | Sylviidae |  | *Sylvia borin* | 16 | 32 | 0.89 | 1.91 | 54.40 | 42 | 0 | 0 | 0 | 13 | 37 |
| Passeriformes | | Turdidae |  | *Turdus merula* | 17 | 88 | 0.84 | 4.55 | 336.34 | 198 | 0 | 0 | 2 | 55 | 597 |
| Passeriformes | | Turdidae |  | *Turdus philomelos* | 18 | 44 | 0.85 | 2.71 | 101.35 | 76 | 0 | 0 | 0 | 58 | 429 |
|  | |  |  |  |  |  |  |  |  |  |  |  |  |  |  |
| Artiodactyla | | Cervidae |  | *Cervus elaphus* | 1 | 17** | 0.70** | 1.49** | 17.73** | 0.79** | 0.15** | 0.05** | 0.01** | 1 | 4 |
| Rodentia | | Gliridae |  | *Dryomys nitedula* | 5 | 17** | 0.70** | 1.49** | 17.73** | 0.79** | 0.15** | 0.05** | 0.01** | 2 | 4 |
| Carnivora | | Mustelidae |  | *Martes martes* | 8 | 17** | 0.70** | 1.49** | 17.73** | 0.79** | 0.15** | 0.05** | 0.01** | 2 | 30‡ |
| Galliformes | | Phasianidae |  | Unknown | 9 | 17** | 0.70** | 1.49** | 17.73** | 0.79** | 0.15** | 0.05** | 0.01** | 1 | 2 |
| Rodentia | | Cricetidae |  | *Myodes glareolus* | 11 | 17** | 0.70** | 1.49** | 17.73** | 0.79** | 0.15** | 0.05** | 0.01** | 2 | 3 |
| Passeriformes | | Prunellidae |  | *Prunella modularis* | 13 | 17** | 0.70** | 1.49** | 17.73** | 0.79** | 0.15** | 0.05** | 0.01** | 1 | 1 |
| Rodentia | | Muridae |  | *Apodemus flavicollis* | 19 | 17** | 0.70** | 1.49** | 17.73** | 0.79** | 0.15** | 0.05** | 0.01** | 0 | 1 |
| Artiodactyla | | Suidae |  | *Sus scrofa* | 20 | 17** | 0.70** | 1.49** | 17.73** | 0.79** | 0.15** | 0.05** | 0.01** | 0 | 0 |
|  | |  |  |  |  |  |  |  |  |  |  |  |  |  |  |
| Artiodactyla | | Bovidae |  | *Bison bonasus* |  | 0 |  |  |  |  |  |  |  | 0 | 1 |
| Columbiformes | | Columbidae |  | *Columba palumbus* |  | 0 |  |  |  |  |  |  |  | 0 | 1 |
| Passeriformes | | Corvidae |  | *Garrulus glandarius* |  | 0 |  |  |  |  |  |  |  | 0 | 3 |
| Rodentia | | Gliridae |  | *Muscardinus avellanarius* |  | 0 |  |  |  |  |  |  |  | 0 | 4 |
| Passeriformes | | Fringillidae |  | *Pyrrhula pyrrhula* |  | 0 |  |  |  |  |  |  |  | 0 | 5 |
| Rodentia | | Sciuridae |  | *Sciurus vulgaris* |  | 0 |  |  |  |  |  |  |  | 0 | 1 |
|  | *Scats with seeds of at least one fleshy-fruited plant species co-occuring with *F. alnus.* We included only those scats in the analyses, which were found at the same time as scats with seeds of *F. alnus* (see methods)*.* ‡*Martes martes* uses scats to mark territories. We included all scat of *M. martes* in the analyses independent of the period of year when we found the scat (see methods). ** Fruit removal and fruit handling of these animals was not observed. We assigned them the mean values of fruit removal and handling probabilities of other rare animals in the seed deposition network (less than ten scats in total; see methods). *Apodemus flavicollis* and *Sus scrofa* were only observed using camera trapping in a pilot study. | | | | | | | | | | | | | | |

Supplementary Figure 1: Life cycle diagram with a pre-reproductive census of the temperate tree *F. alnus*. Large and small circles display established individuals and new recruits.

Breakage

**Census *t***

**Census *t+1***

Reproduction

Survival

No breakage

Growth

Resprout

Seedling
recruitment

Growth

Dispersal

n (z’,c’,t+1)

n (z,c,t)


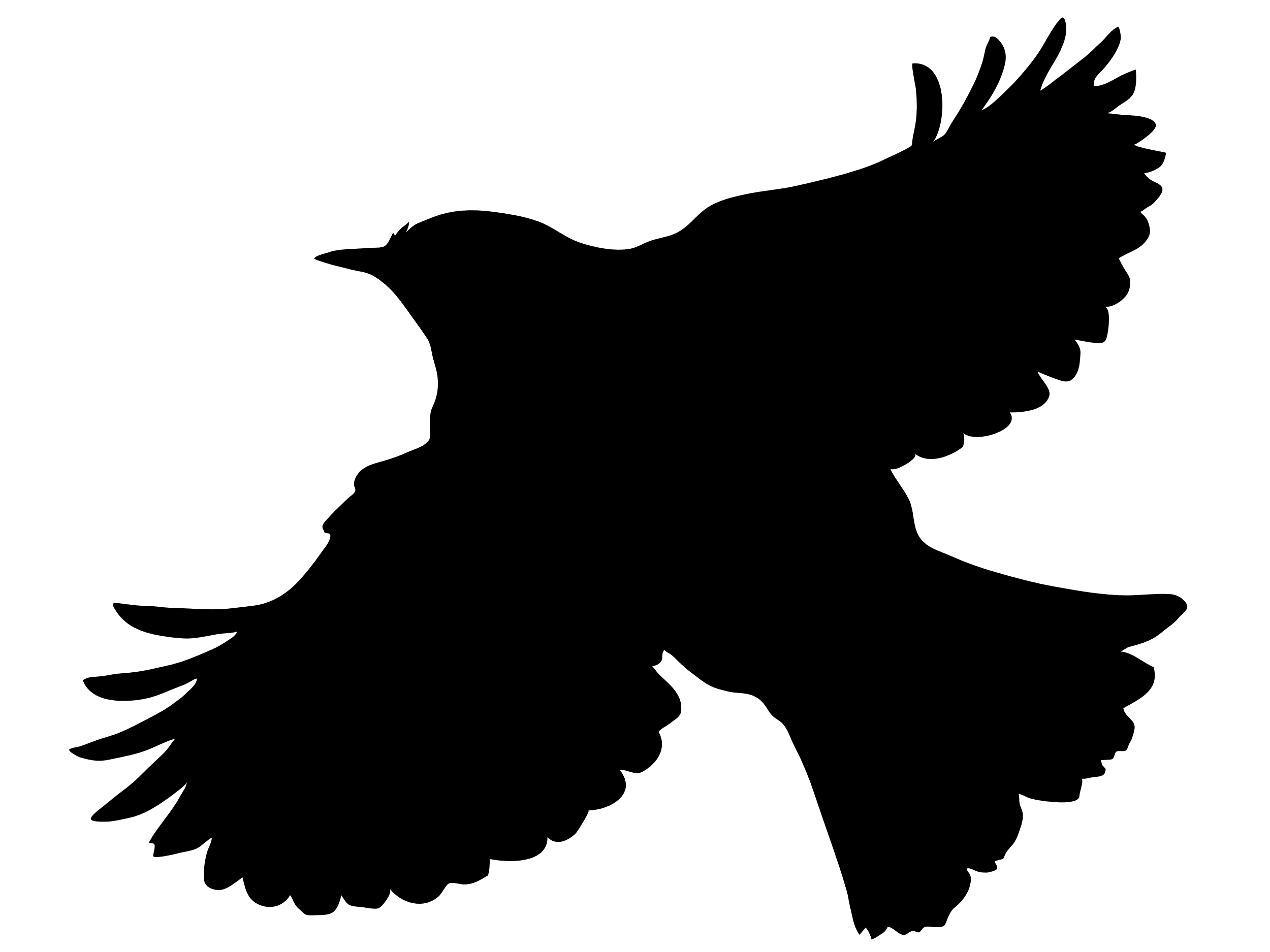


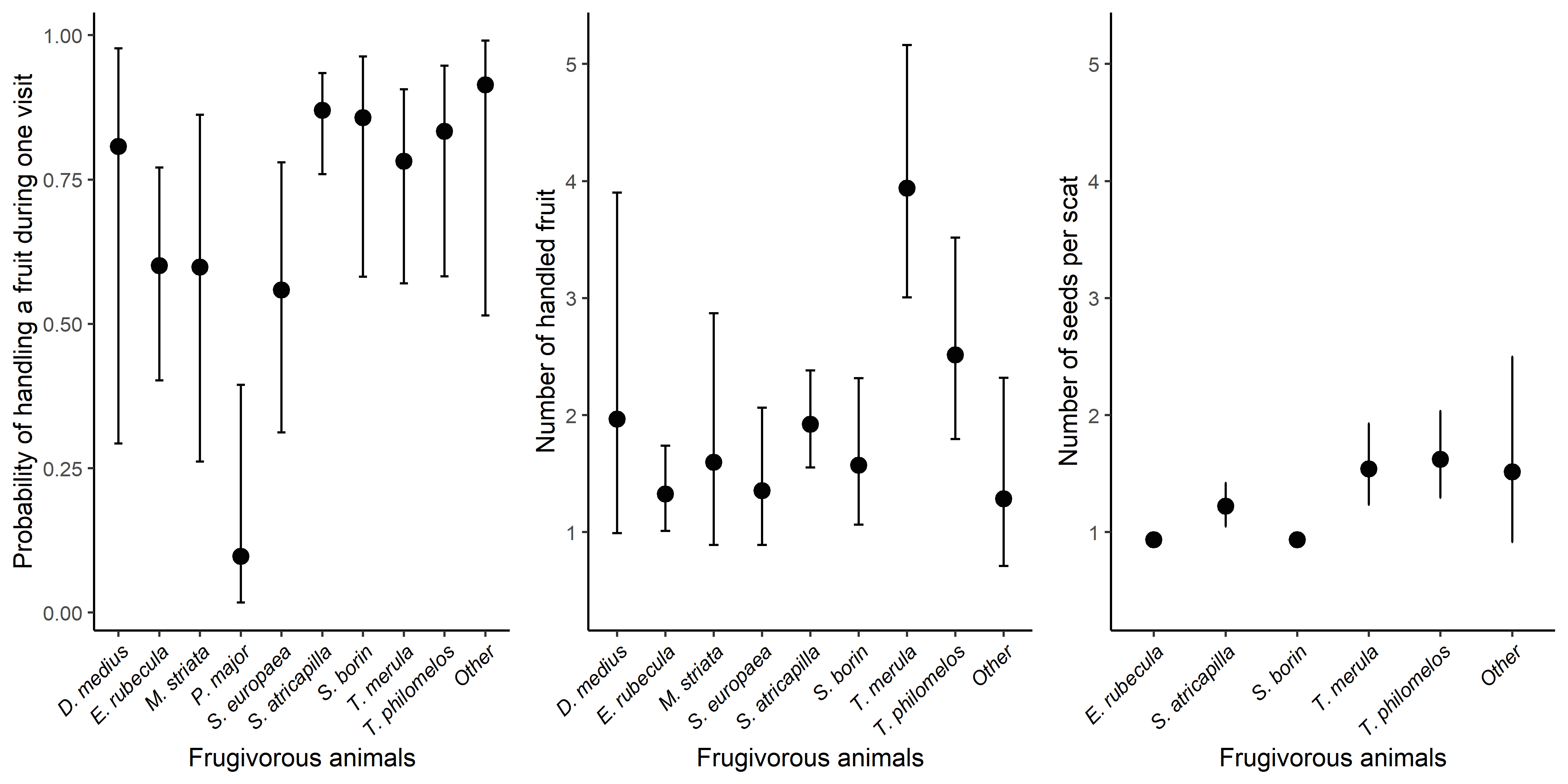
Supplementary Figure 2: (a) The probability of handling a fruit, (b) the number of handled fruit during each visit and (c) the number of seeds per scat as an effect of animal species. The numbers in the brackets reflect the species labels in the interaction network (see manuscript, Figure 1). Mean ± 95% CI.

The effect of animals on the probability of handling a fruit during one visit (Wald-χ² = 48.58, p < 0.001), number of fruits (Wald-χ² = 78.69, p < 0.001) was significant, and on number of seeds per scat (Wald-χ² = 10.63, p = 0.059) marginally significant.

Here (only in Supplementary Figure 2), we analysed the effect of frugivorous animal species on different components of fruit removal with generalised linear mixed models with site and observer as random factors. The data on fruit removal is from ^1^ and on seed deposition from ^2^. To analyse the effect of frugivore species identity on the probability of handling a fruit during a visit to *F. alnus*, we used a logit link and a binomial error distribution. To analyse the effect of frugivore species identity on the number of fruits handled during each of these visits, we used a log link and a Poisson error distribution. In the analysis on the effect of frugivore species on the number of seeds per scat, we used site as a random factor, frugivore species as a fixed factor, and a log link and a Poisson error distribution. In this model, we excluded a single outlier because it affected the model outcome, and included the scat ID as observation-level random effect to account for overdispersion.

Notably, in the manuscript we used bootstrapping of the raw data to create uncertainty in fruit removal, handling and seed deposition.


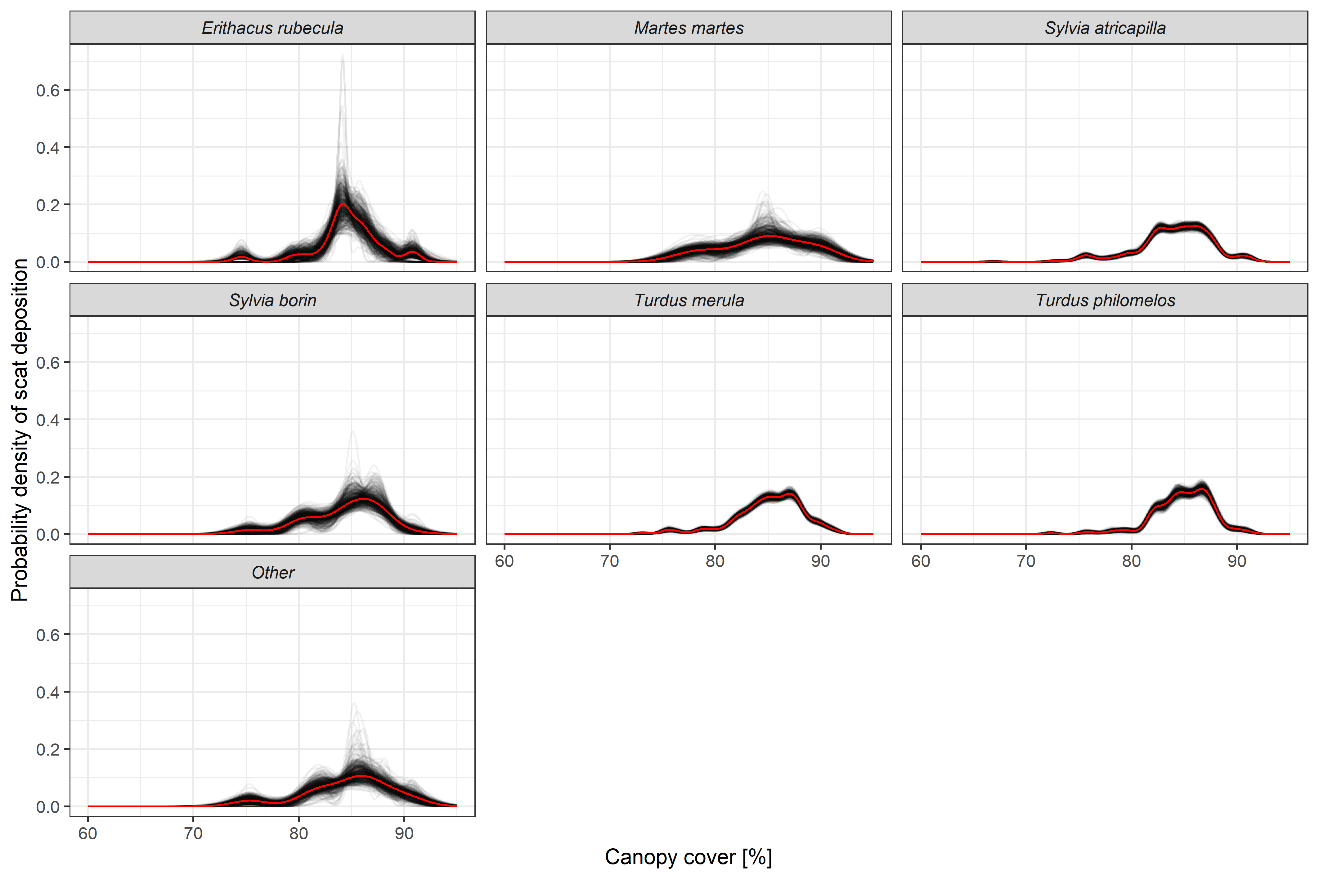
Supplementary Figure 3: Uncertainty in the relative deposition pattern of animal species along the canopy cover gradient in Białowieża Forest, Eastern Poland. Animal disperser species were *Erithacus rubecula, Martes martes, Sylvia atricapilla, Sylvia borin, Turdus merula, Turdus philomelos* and all remaining disperser species (‘Other’). The red solid line displays the mean deposition pattern. Each grey line displays one of 500 draws from non-parametric bootstraps with replacement.


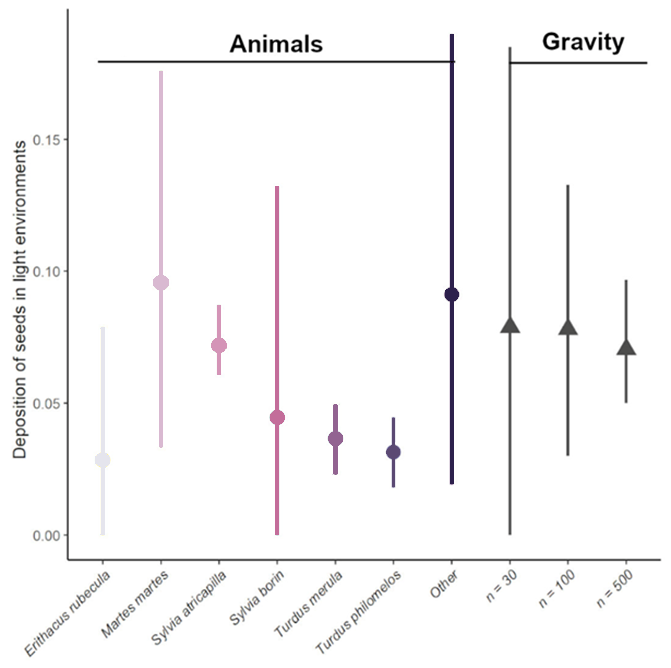
Supplementary Figure 4: Uncertainty in the relative deposition pattern of seeds by animal species or gravity into the 50% lightest environments (forest gaps, edges) along the canopy cover gradient in Białowieża Forest, Eastern Poland. Animal disperser species were *Erithacus rubecula, Martes martes, Sylvia atricapilla, Sylvia borin, Turdus merula, Turdus philomelos* and all remaining disperser species (‘Other’). Seed deposition of gravity dispersal was modelled as the relative abundance of microhabitats along the canopy gradient in the forest depending on the number of replicates for each bootstrap, with only 30 replicates (similar to *E. rubecula*, *Martes martes*, *Sylvia borin* and ‘Other’ disperser), 100 replicates and 500 replicates (similar to *S. atricapilla, T. merula* and *T. philomelos*). The results of gravity thus correspond to what we expected based on random deposition of seeds by animals. Mean ± 95% prediction intervals based on 500 non-parametric bootstraps.

Supplementary Discussion 1: Seedling recruitment of *Frangula alnus*

To analyze the effect of canopy cover and year on seedling recruitment in *F. alnus*, we used generalized linear mixed models with the number of seedlings that had recruited in spring and the number of non-recruited seeds in the same plot as a response variable. We included the plots within sites as a random factor. In these models, we used a logit link and a beta-binomial error distribution to account for overdispersion.

Peak recruitment of *F. alnus* was low in the Białowieża Forest and was only influenced by year (χ² = 11.32, p = 0.004, Fig S5a), but not canopy cover (χ² = 0.69, p = 0.407) and the interaction of year and canopy cover (χ² = 0.46, p = 0.795). Only one seedling recruited from seeds sown in 2017.

On average, the probability of recruitment of *F. alnus* was 0.844% in the first year, 0.002% in the second year and 0.041% in the third year after dispersal. Because seedling recruitment in the second and third year was negligible, we modelled seedling recruitment of *F. alnus* without persistent seed bank.

In 2018, we additionally sowed out 500 seeds of *F. alnus* beneath 20 conspecific adults in the forest. After correcting for naturally occurring seedlings, the conspecific adults did not affect seedling recruitment of *F. alnus* in the next year (χ² = 0.29, p = 0.588, Supplementary Figure 5b).


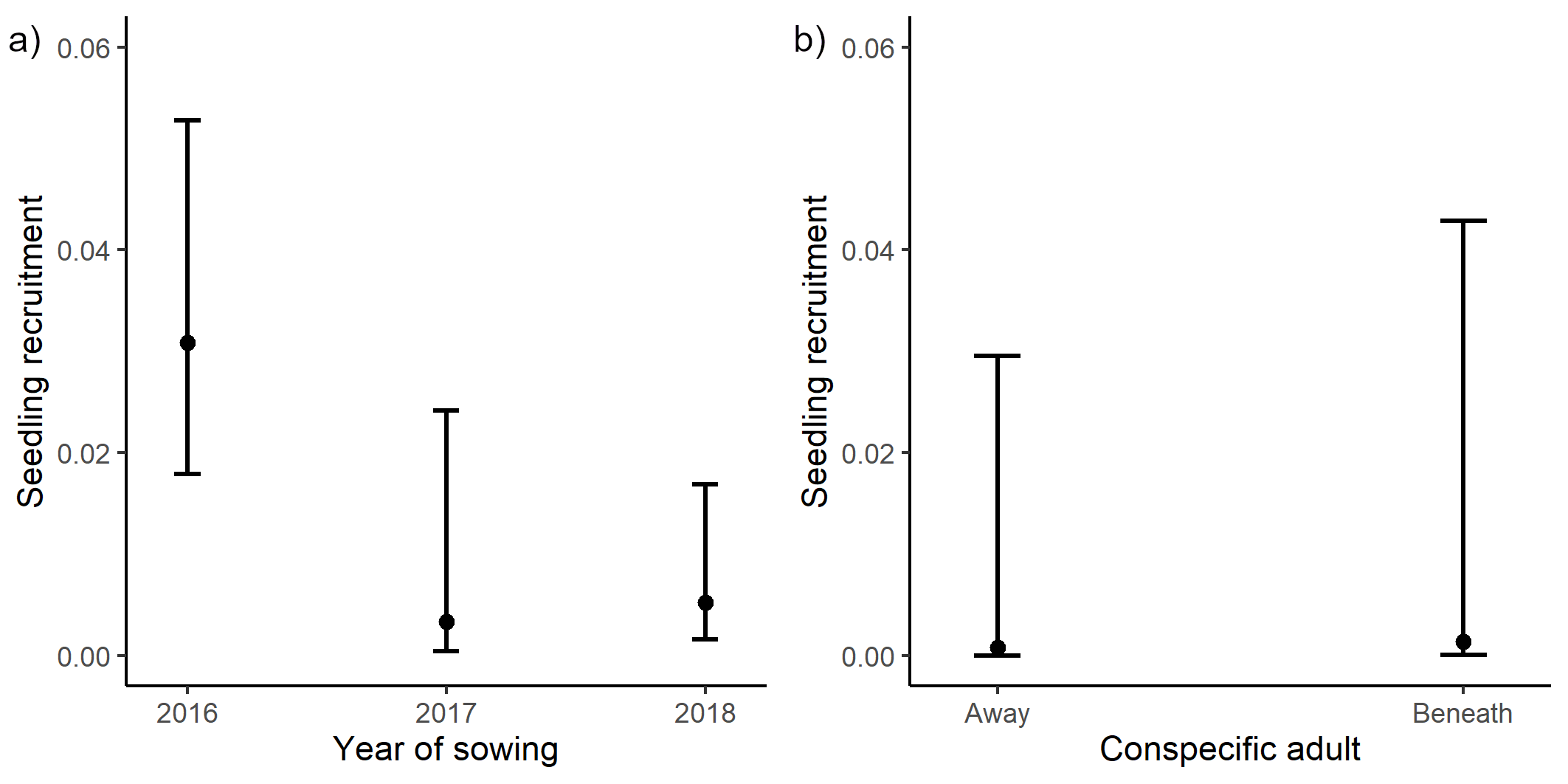
**Supplementary Figure 5:** (a) Effects of the year of sowing and (b) conspecific adults on recruitment of seedlings of *Frangula alnus* in the Białowieża Forest. The results are shown for the year of peak recruitment of *F. alnus* (after one year). Mean ± 95%CI.

Supplementary Table 4: Summary of the rank selection of the most parsimonious model across the vital rates (survival, growth, fruiting probability and number of fruits) of *F. alnus* in Białowieża Forest.

| **Model** | **Fixed factors** | **Survival** | | | **(log) Growth** | | | **Fruiting prob.** | | | **Number of fruits** | | |  | **Summed ranks** | **Mean dAICc** |
| --- | --- | --- | --- | --- | --- | --- | --- | --- | --- | --- | --- | --- | --- | --- | --- | --- |
|  |  | Rank | AICc | dAICc | Rank | AICc | dAICc | Rank | AICc | dAICc | Rank | AICc | dAICc |  |  |  |
| 1 | Year (Y) + Canopy cover (C) +  Diameter (D) + Diameter² (D²) | **1** | **687.4** | **0.0** | 6 | -1484.7 | 10.2 | 5 | 842.2 | 3.4 | 6 | 5660.4 | 32.4 |  | 18 | 11.5 |
| 2 | Y * C + D + D² | 2 | 689.3 | 1.9 | 7 | -1483.6 | 11.3 | 7 | 843.4 | 4.6 | 7 | 5663.0 | 35.0 |  | 23 | 13.2 |
| **3** | **C + Y * D + Y * D²** | 4 | 690.7 | 3.4 | **1** | **-1494.9** | **0.0** | **1** | **838.8** | **0.0** | **1** | **5628.0** | **0.0** |  | **7** | **0.8** |
| 4 | Y + C * D + C * D² | 3 | 690.6 | 3.3 | 8 | -1481.5 | 13.4 | 6 | 842.7 | 3.9 | 8 | 5663.8 | 35.8 |  | 25 | 14.1 |
| 5 | Y * C + Y * D + Y * D² | 5 | 692.7 | 5.4 | 2 | -1493.8 | 1.0 | 3 | 839.9 | 1.1 | 2 | 5632.0 | 4.0 |  | 12 | 2.9 |
| 6 | Y * D + Y * D² + C * D + C * D² | 6 | 694.0 | 6.6 | 3 | -1491.3 | 3.6 | 2 | 839.7 | 0.9 | 3 | 5636.2 | 8.2 |  | 14 | 4.8 |
| 7 | Y * C + Y * D + Y * D² + C * D + C * D² | 7 | 696.0 | 8.7 | 4 | -1490.3 | 4.6 | 4 | 841.8 | 3.0 | 4 | 5636.2 | 8.2 |  | 19 | 6.1 |
| 8 | Y * C * D + Y * C * D² | 8 | 698.9 | 11.5 | 5 | -1481.5 | 13.4 | 8 | 844.7 | 5.9 | 5 | 5663.8 | 35.8 |  | 26 | 16.6 |
| AICc, Akaike's information criterion with a correction for small sample sizes. | | | | | | | | | | | | | | | | |
| The diameter of plant individuals was log10-transformed, and canopy cover as well as diameter were standardized before analyses. | | | | | | | | | | | | | | | | |
| All component models included a quadratic term of diameter (“Diameter²”) as a fixed factor and study site as a random factor. The number of fruits also included an observation-level random effect to account for overdispersion. | | | | | | | | | | | | | | | | |
| The best-ranked model of a vital rate based on AICc is bold. | | | | | | | | | | | | | | | | |

Supplementary Table 5: Analyses of variance of the effects of year, canopy and diameter on plant vital rates (survival probability, diameter next, fruiting probability, number of fruits, breakage probability and resprouting) of *F. alnus* in Bialowieza Forest, Poland.

|  | **Survival probability** | | |  | **Diameter next** | | |  | **Fruiting probability** | | |
| --- | --- | --- | --- | --- | --- | --- | --- | --- | --- | --- | --- |
|  | df | Wald-χ² | p |  | df | Wald-χ² | p |  | df | Wald-χ² | p |
| Canopy cover | 1 | 5.2 | **0.023** |  | 1 | 8.8 | **0.003** |  | 1 | 6.0 | **0.014** |
| Year | 1 | 10.0 | **0.002** |  | 1 | 104.0 | **< 0.001** |  | 2 | 35.6 | **< 0.001** |
| Diameter | 1 | 38.4 | **< 0.001** |  | 1 | 25162.4 | **< 0.001** |  | 1 | 122.3 | **< 0.001** |
| Diameter² | 1 | 27.2 | **< 0.001** |  | 1 | 18.1 | **< 0.001** |  | 1 | 31.2 | **< 0.001** |
| Year * Diameter | 1 | 0.5 | 0.464 |  | 1 | 7.5 | **0.006** |  | 2 | 3.9 | 0.144 |
| Year * Diameter² | 1 | 0.5 | 0.476 |  | 1 | < 0.1 | 0.996 |  | 2 | 7.2 | **0.027** |
| Residual | 1094 |  |  |  | 900 |  |  |  | 1786 |  |  |
|  |  |  |  |  |  |  |  |  |  |  |  |
|  | **Number of fruits** | | |  | **Breakage probability** | | |  | **Resprouting next** | | |
|  | df | Wald-χ² | p |  | df | Wald-χ² | p |  | df | Wald-χ² | p |
| Canopy cover | 1 | 4.6 | **0.031** |  |  |  |  |  |  |  |  |
| Year | 2 | 9.1 | **0.011** |  |  |  |  |  |  |  |  |
| Diameter | 1 | 61.4 | **< 0.001** |  | 1 | 9.5 | **< 0.001** |  | 1 | 133.6 | **< 0.001** |
| Diameter² | 1 | 5.5 | **0.020** |  |  |  |  |  | 1 | 28.1 | **< 0.001** |
| Year * Diameter | 2 | 0.6 | 0.736 |  |  |  |  |  |  |  |  |
| Year * Diameter² | 2 | 6.6 | **0.038** |  |  |  |  |  |  |  |  |
| Residual | 514 |  |  |  | 1883 |  |  |  | 8 |  |  |
| Diameter, diameter of plant individuals at time t; Diameter next, diameter of plant individuals at time t+1; Resprouting next, diameter of plant individuals after breakage. | | | | | | | | | | | |


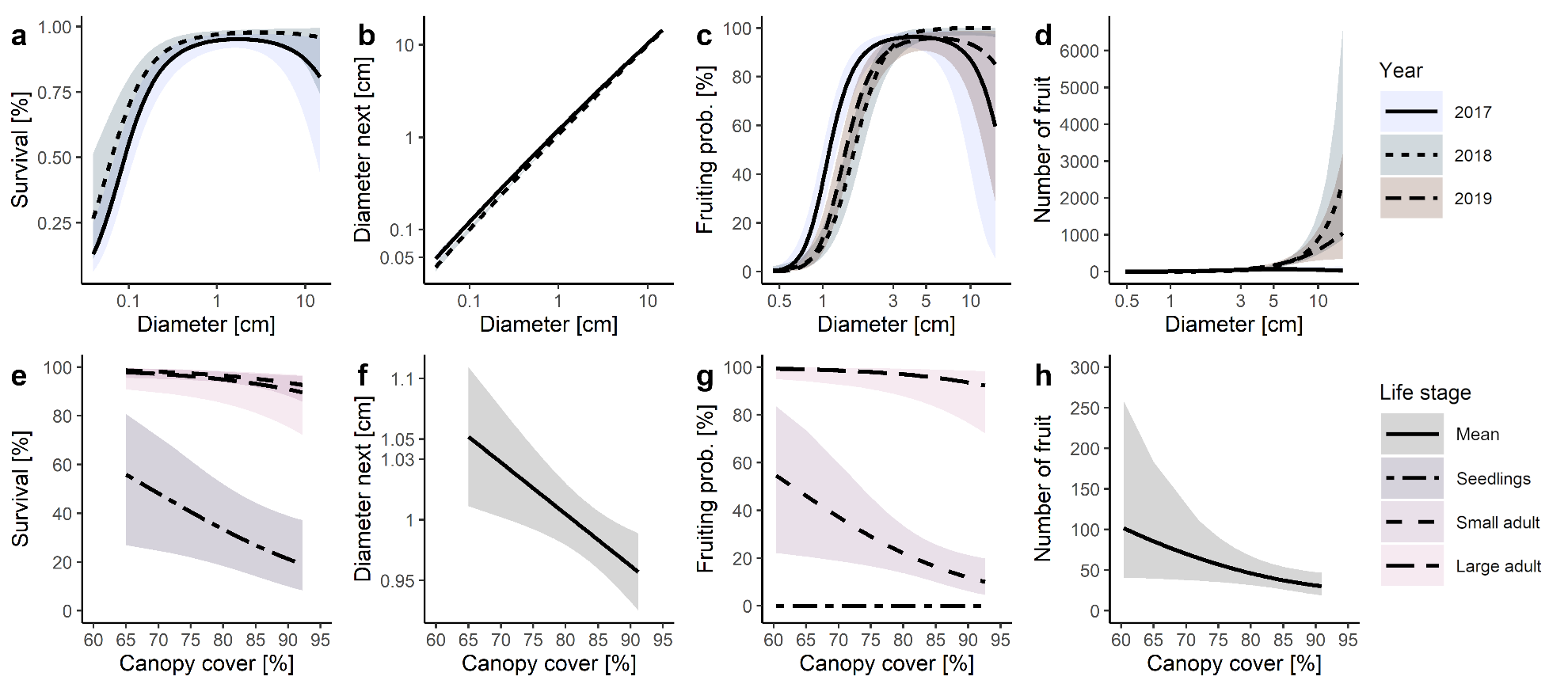
Supplementary Figure 6: The effect of (a,b,c,d) diameter of plant individuals and study year (2017, 2018, 2019) and (e, f, g, h) canopy cover on the (a,d) probability to survive from time t to t+1, (b,f) next diameter at time t+1, (c,g) probability to fruit and (d,h) the number of fruit of *F. alnus* in Białowieża Forest, Eastern Poland. Due to the non-linear relationships, we illustrated the effect of canopy cover on survival and fruiting probability for three life stages (seedlings, small adult, large adult). Predicted mean ± 95%CI. Note the log-scale for diameter and diameter next.


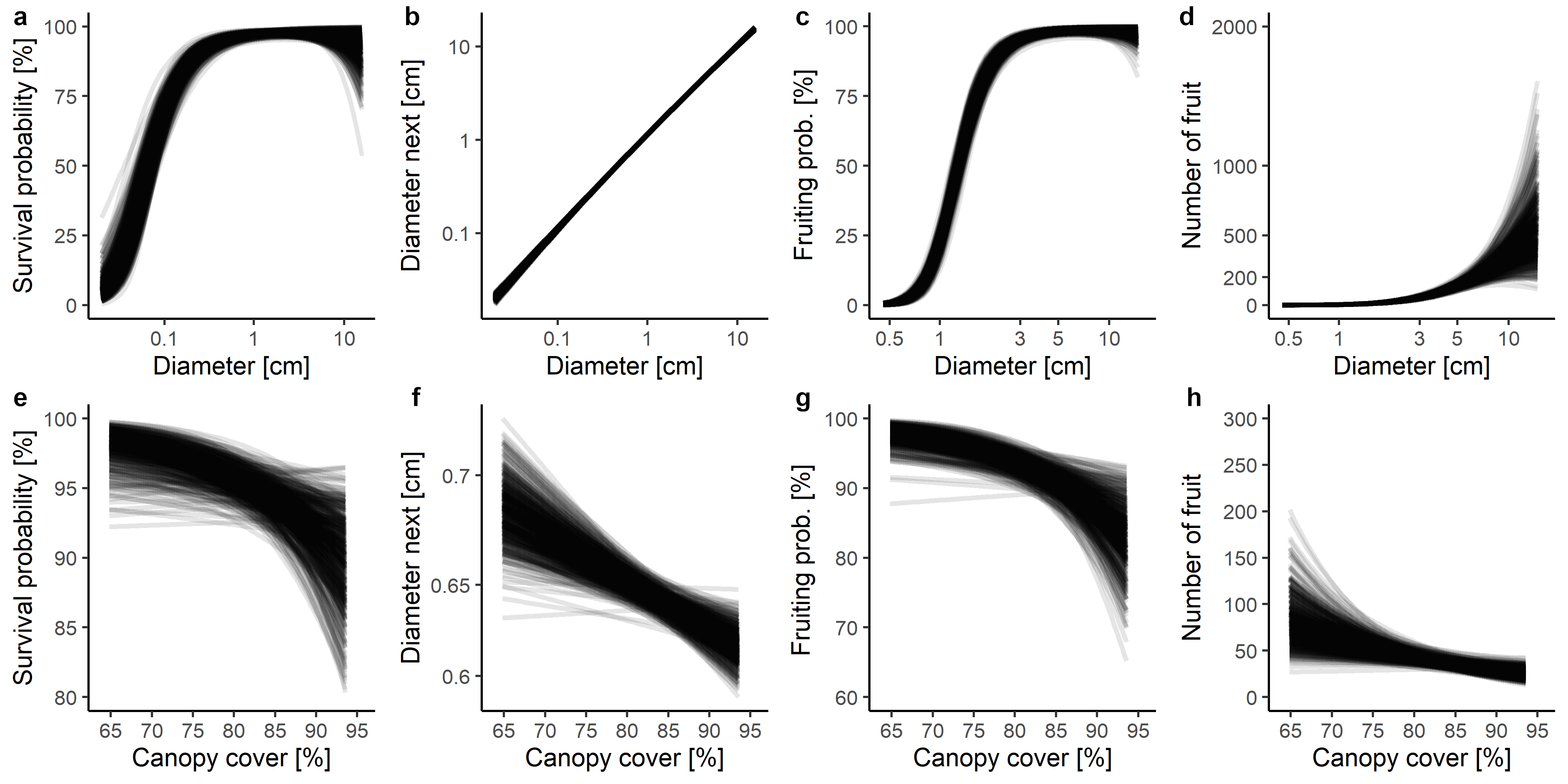
Supplementary Figure 7: The effect of (a,b,c,d) diameter of plant individuals averaged across study years (2017, 2018, 2019) and (e, f, g, h) canopy cover on the (a,d) probability to survive from time t to t+1, (b,f) next diameter at time t+1, (c,g) probability to fruit and (d,h) the number of fruit of *F. alnus* in Białowieża Forest, Eastern Poland. Each grey line displays one of 500 draws from non-parametric bootstraps with replacement, collectively representing uncertainty in the vital rate regressions. Note the log-scale for diameter and diameter next.


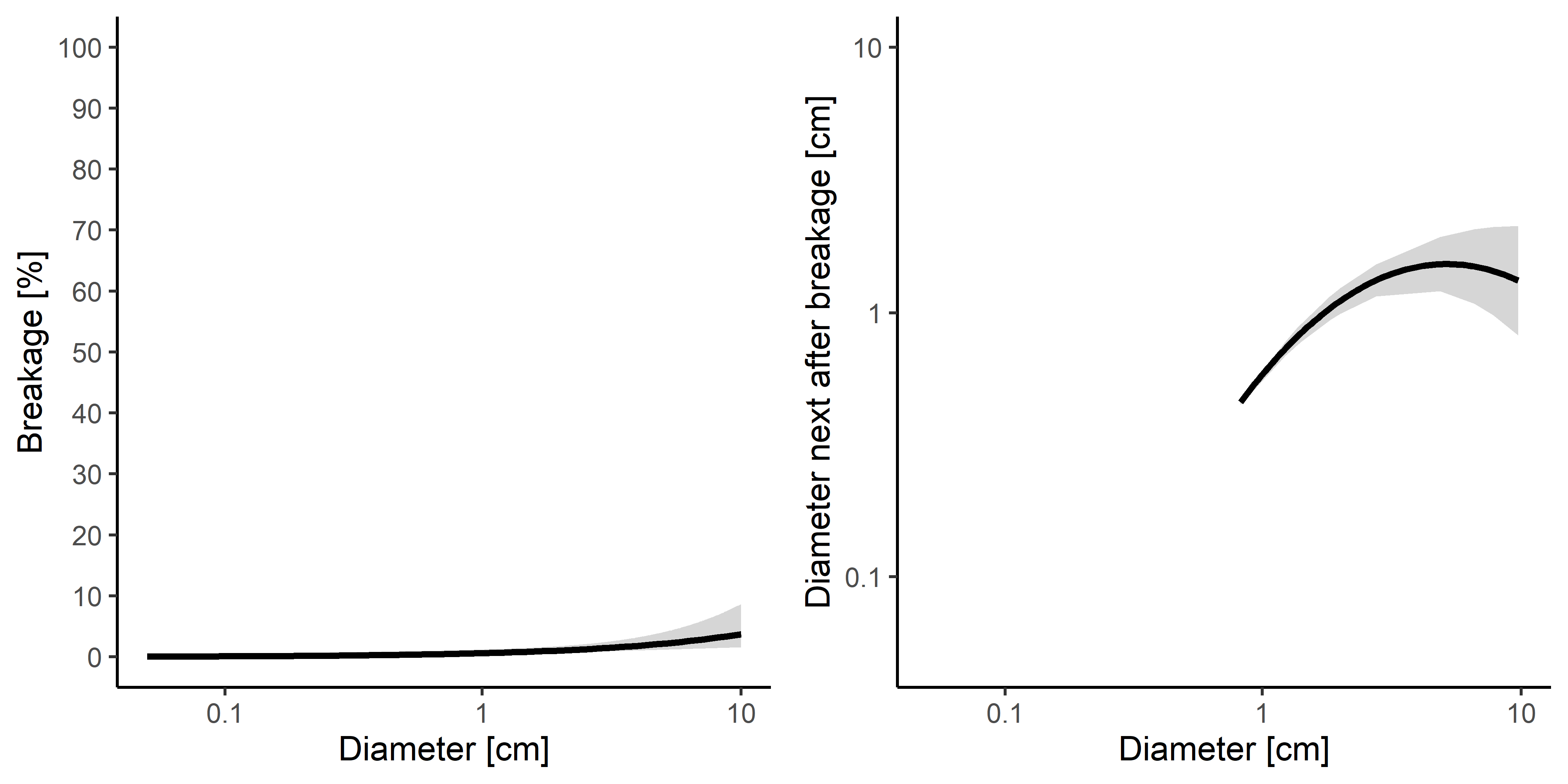
Supplementary Figure 8: The effect of diameter of plant individuals on (a) the probability to break and survive from time t to t+1, (b) the diameter of individuals after breakage at time t+1 of *F. alnus* in Białowieża Forest, Eastern Poland. Predicted mean ± 95%CI. Note the log-scale for diameter and diameter next after breakage.


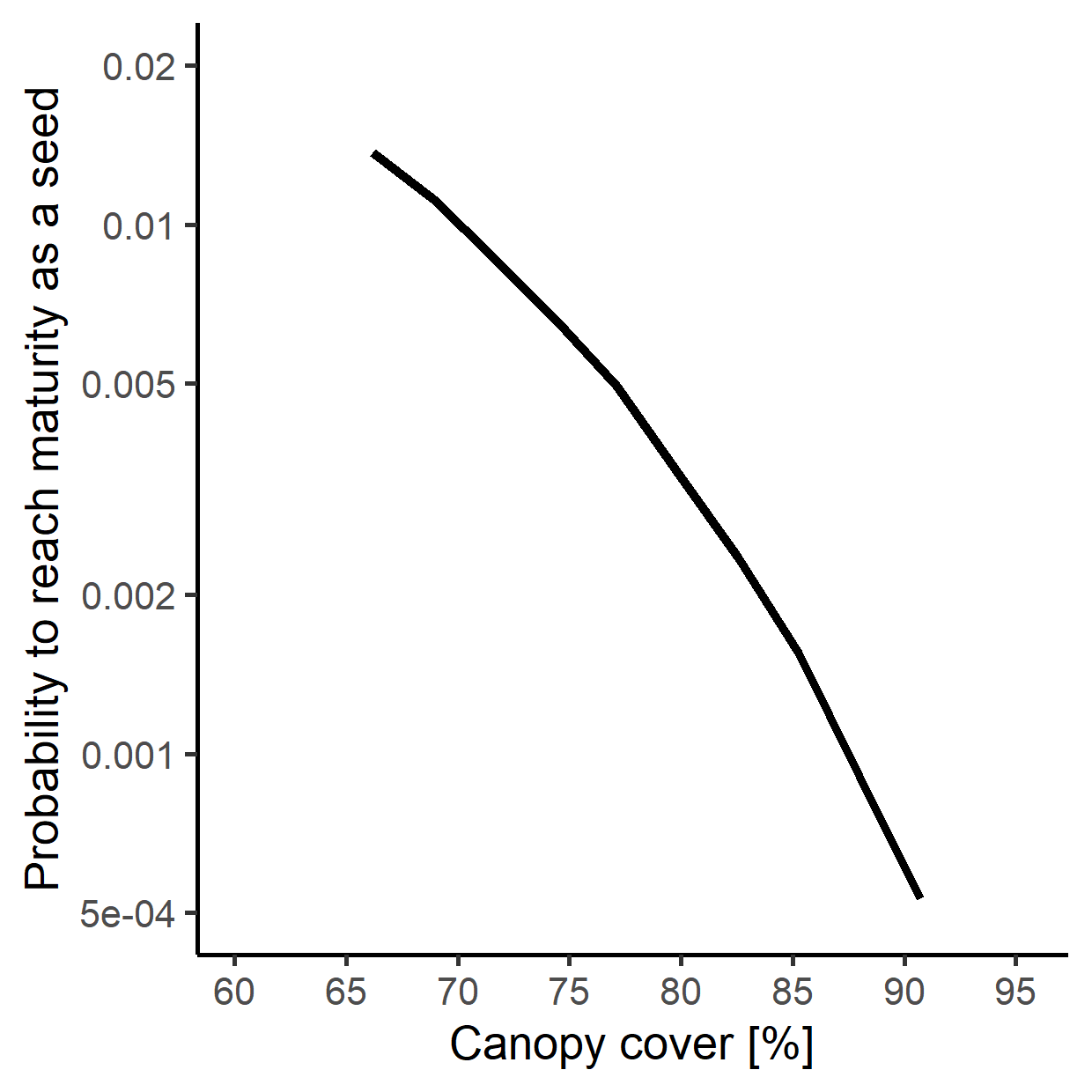
Supplementary Figure 9: The probability of a seed of *F. alnus* to reach adulthood as an effect of canopy cover in Białowieża Forest, Eastern Poland. Note the log-scale for the probability.


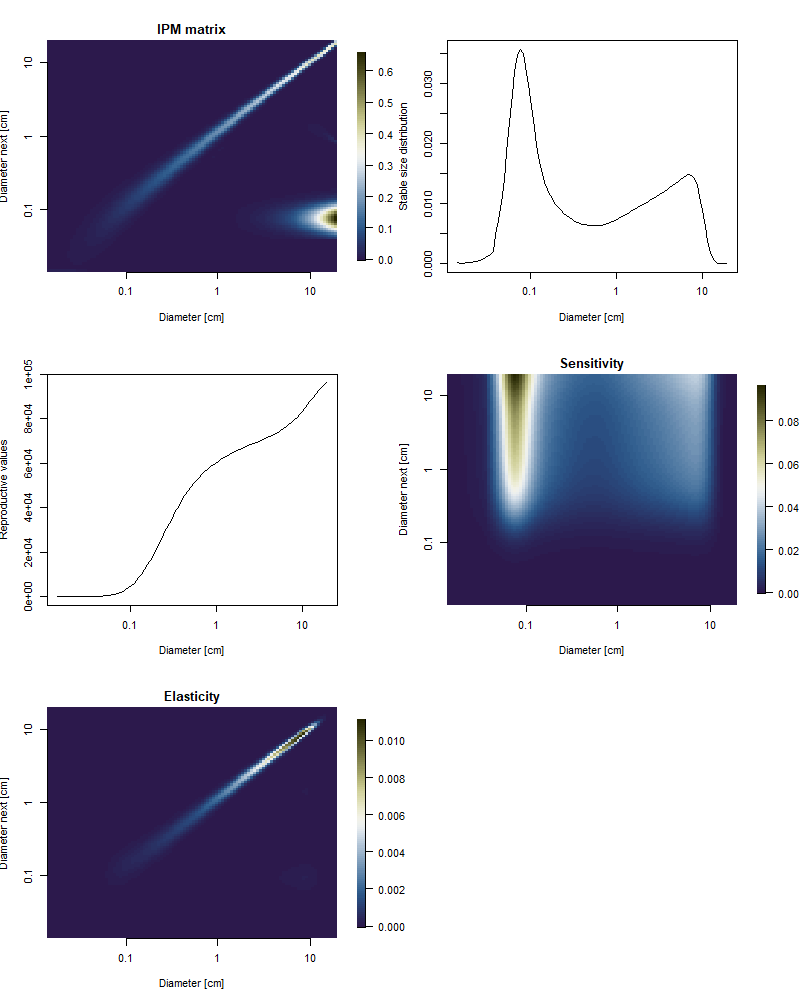
Supplementary Figure 10: Basic model output of the meta-population of *F. alnus* with a fecundity kernel modelling gravity dispersal in Białowieża Forest, Eastern Poland. (a) IPM matrix, (b) stable size distribution, (c) reproductive value of individuals, (d) sensitivity and (e) elasticities of the IPM matrix. Note the log-scale for diameter and diameter next.


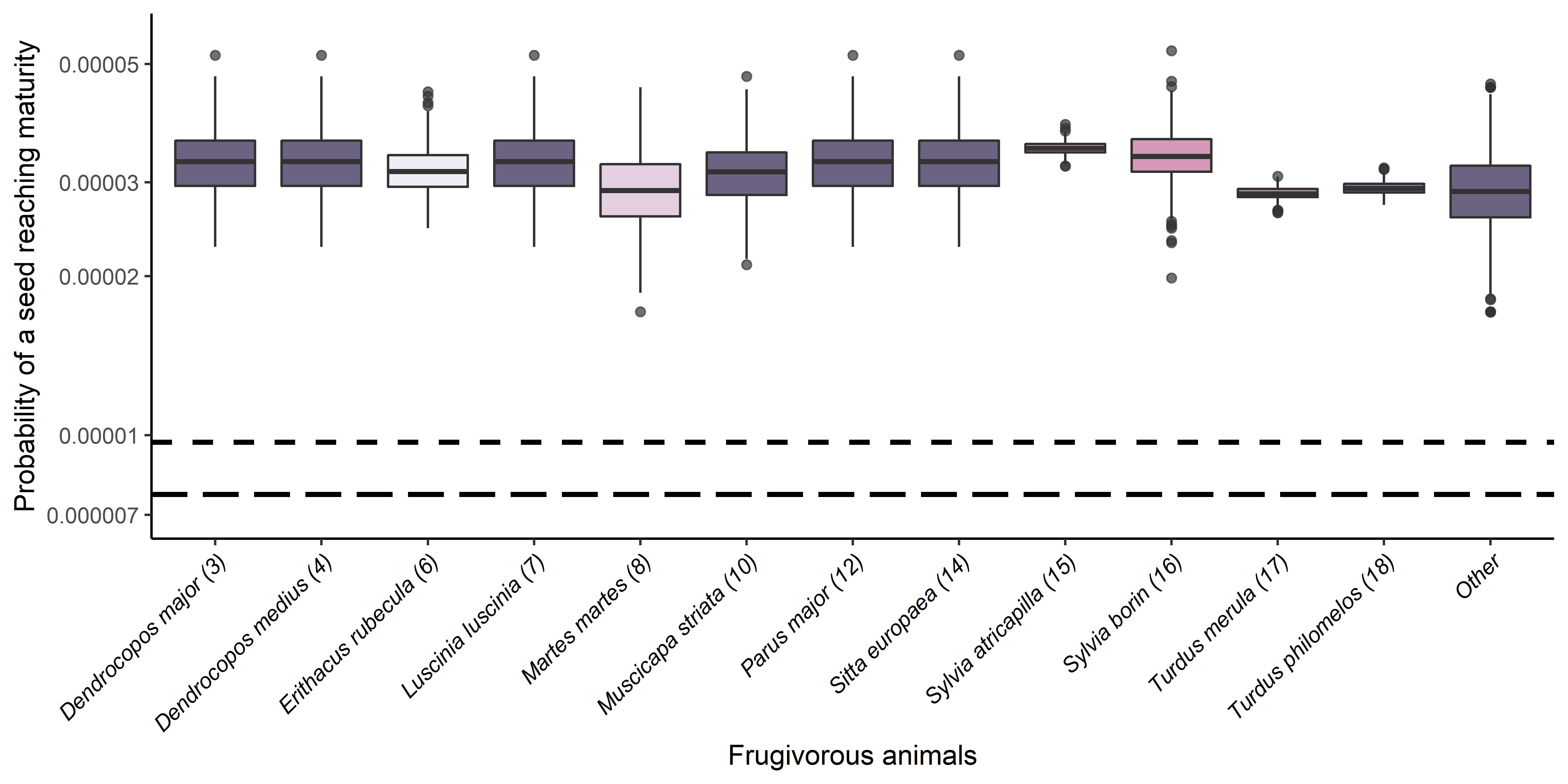
Supplementary Figure 11: Boxplots of the effect of legitimate disperser species on the quality of seed dispersal (i.e. the probability of a seed reaching maturity) of *F. alnus* in Białowieża Forest, Eastern Poland. Uncertainty in the probability of a seed reaching maturity is based on 500 non-parametric bootstrap of only animal-related seed dispersal components; plant-related components of the model (e.g. uncertainty in vital rates or the effect of canopy cover on vital rates) were kept constant. Horizontal lines depict the quality of seed dispersal by gravity, when *F. alnus* is fully established along the entire canopy cover gradient in the forest (‘Gravity dispersal’), or is established only along the 50%-darkest part of the gradient, i.e. *F. alnus* depends on animal seed dispersal to forest gaps or edges (‘Gravity dispersal with disturbance events’). Please note the log-scale for the probability of a seed reaching maturity. While the reduced probability of a seed reaching maturity in *Martes martes* and the ‘other’ disperser (including small rodents) is due to low rates of seed predation (~ 5%, see Supplementary Table 3), the reduced probability of a seed reaching maturity in *Turdus merula* and *T. philomelos* is due to the non-random deposition of seeds into dark environments (see Supplementary Figure 4). The seed-predating bird species *Coccothraustes coccothraustes* was not included.


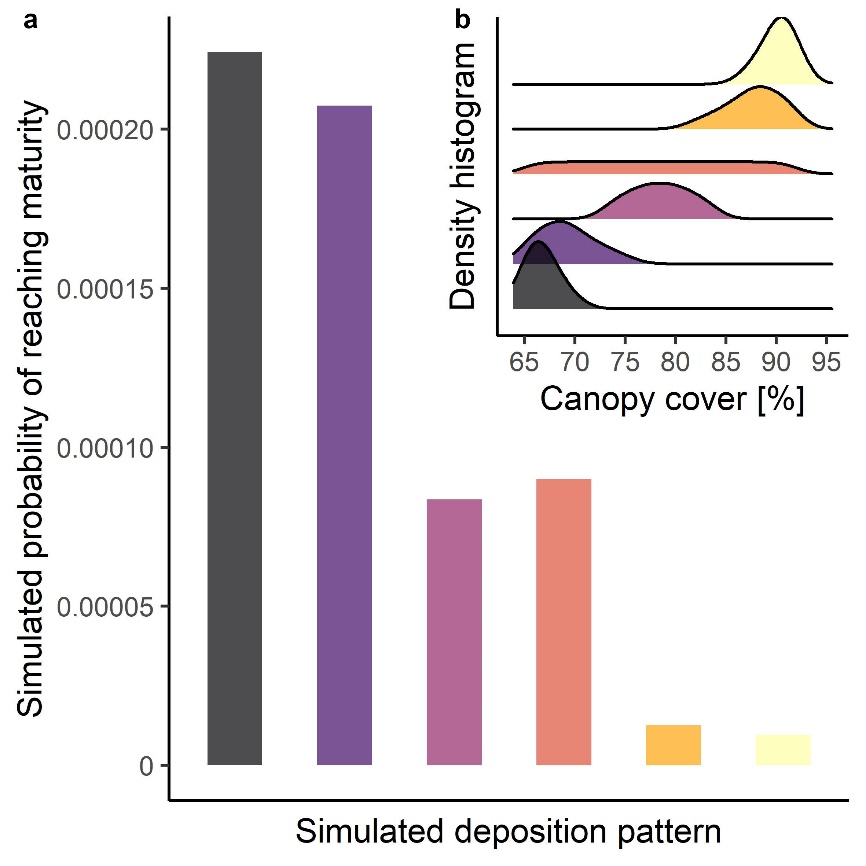
Supplementary Figure 12: Simulations of (a) probabilities for seeds of *F. alnus* to reach adulthood, when all parameters are constant except the underlying (b) patterns of seed deposition. In (a) *S. atricapilla* was chosen as the baseline disperser.

Supplementary Discussion 2: Seed deposition beneath conspecific adults


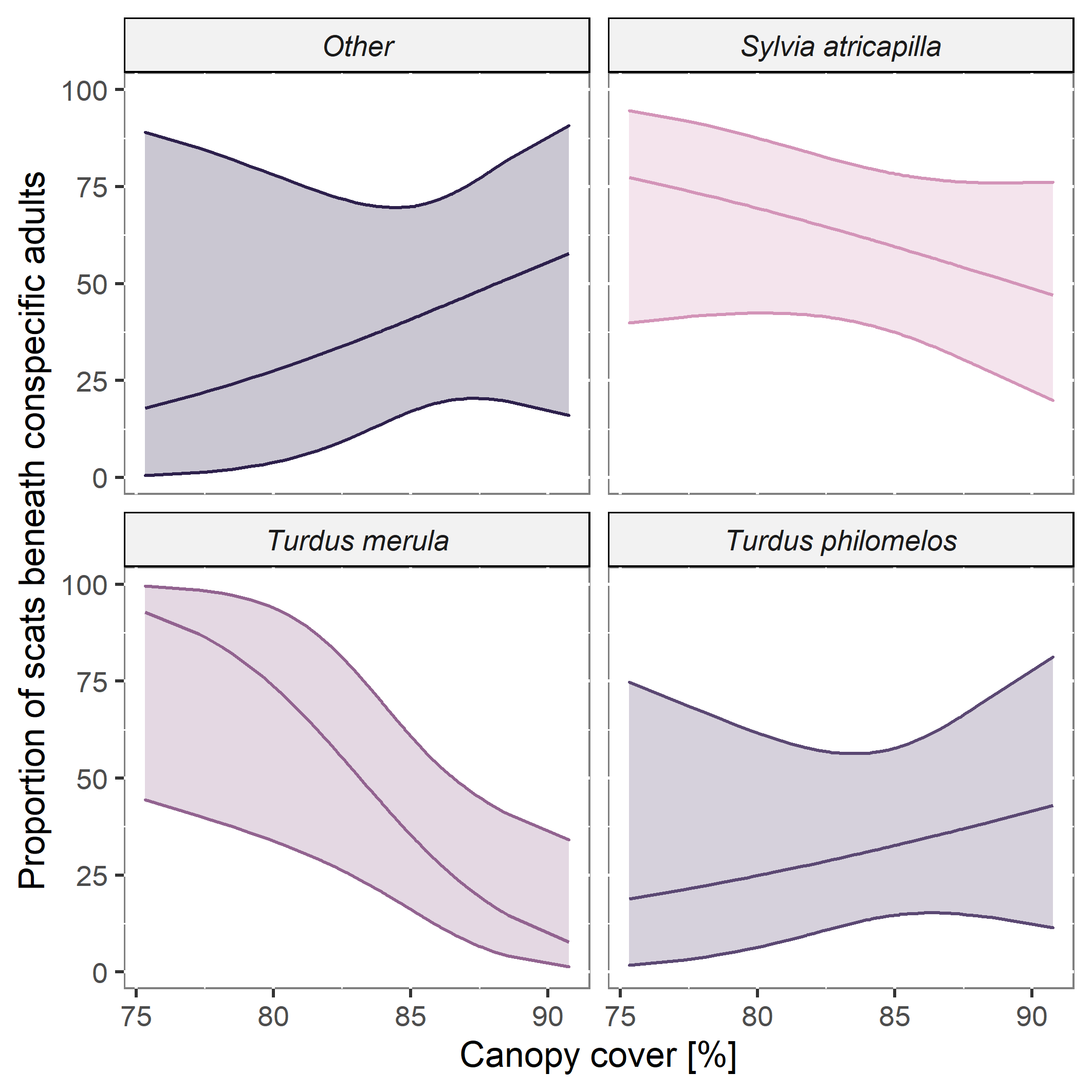
We found 63.27% of all scats with seeds of *Frangula alnus* beneath fruiting conspecific adults. Seeds of *F. alnus* were deposited more often beneath conspecific adults by *Sylvia atricapilla* (60%, 95%CI: 38-79%) than by *T. philomelos* (32%, 14-57%) or the remaining disperser (“other”, 40%, 16-70%) (Wald-χ² = 14.01, p = 0.003). There was a weak interaction between animal disperser and canopy cover (Wald-χ² = 7.06, p = 0.070, Supplementary Figure 12). In light environments, *T. merula* almost exclusively deposited seeds beneath conspecific adults of *F. alnus*, but the probability of depositing seeds beneath conspecific adults decreased strongly at canopy covers over 80% (Supplementary Figure 12). The seed deposition by other frugivores did not change with canopy cover.

Supplementary Figure 13: The effects of disperser on the proportion of scats with seeds of *Frangula alnus* deposited beneath fruiting conspecific adults along a canopy gradient in the Białowieża Forest. Mean ± 95%CI. Number of scats: Other: n = 30; *Sylvia atricapilla* n = 232; *Turdus merula* n = 55; *Turdus philomelos* n = 58.

Supplementary Discussion 3: No transition of canopy cover in the spatial IPM

The canopy cover gradient of forests is dynamic and changes from one year to the next in natural forests ^3^. We aimed at including natural changes in the canopy structure of the forest into the demographic models of *F. alnus*, but we were ultimately limited for two major reasons: firstly, the period when a hemispherical photo was taken affected the canopy cover of the forest (Wald- *χ*² = 211.14, p < 0.001; Supplementary Figure 14a). In September/October, plants had already discarded some of the leaves why the forest was more open in September/October than in June/July. Secondly, uncertainties in the exact position where the hemispherical photo had been taken resulted in weak correlations of canopy covers over time due to local differences in the surrounding vegetation (Supplementary Figure 14b/c). The relationship between canopy covers were always positive. This shows that light environments were on average lighter and dark environments on average darker despite the underlying variation. Therefore, we believe that the averaged values of canopy covers of up to six photos per transect segment is a good predictor for the actual light availability of a location at ground level.


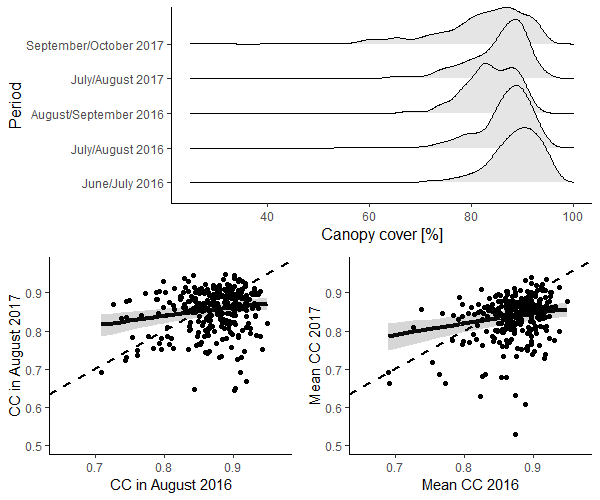


Figure S14: (a) The distribution of canopy cover along the transects segments depending on the period of time when a hemispherical photo was taken in Białowieża Forest. (b/c) Canopy cover of the transect segments over time compared between (b) August in 2016 and 2017 or (c) the years 2016 and 2017 on average. CC = canopy cover. Mean ± 95%CI.

Supplementary Methods 1: Calculating the survival probability of a seed until the age of first reproduction

To calculate the survival probability of a seed reaching adulthood, we used a Markov chain, in which reproduction is an absorbing state in addition to mortality ^4^:

$P_{0}(z^{'}, z,c)=\int_{L}^{U} f_{repr}\left( z,c \right)S\left( z \right)\left( 1-B\left( z \right) \right)G\left( z^{'},z,c \right)n_{t}\left( z,c \right)dzdc+\int_{L}^{U} f_{repr}\left( z,c \right)S\left( z \right)B(z)R\left( z^{'},z \right)n_{t}\left( z,c \right)dzdc$ (Eq 1).

The kernel $P_{0}$ is a modified survival-growth kernel, in which reproduction can be interpreted as a second kind of ‘death’. Similar to the manuscript, here, $P_{0}(z^{'}, z,c)$ growth is modelled for individuals that did and did not break: $S\left( z,c \right)$ models survival, $G\left( z^{'},z,c \right)$ models growth of individuals that did not break with the probability $1-B\left( z \right)$, and the size distribution $R\left( z^{'},z \right)$ of individuals that broke with the probability $B\left( z \right)$, conditional on individual plant size $z$ and canopy $c$.

The fundamental operator $N_{0}$ of the modified reproduction-survival-growth Kernel is given by

$N_{0}(z^{'},z,c)={(I- P_{0}(z^{'},z,c))}^{-1}$ (Eq 2)

and gives the distribution function for expected total time in state $z^{'}$ prior to either dying or reproducing, conditional on initial size $z$ and canopy $c$. Now, we can calculate the probability that an individual reproduces at least once, which is

$B(z^{'},c)=f_{repr}(z,c)N_{0}(z^{'},z,c)$ (Eq 3).

The modified chain $P_{0}$ has two absorbing states, reproduction and death. To compute the mean age at reproduction (for those which reproduce), we computed the transition probability conditional on absorption into only reproduce. The conditional survival kernel is

$P_{repr}\left( z^{'}, z,c \right)=P_{0}\left( z^{'}, z,c \right)*\frac{B\left( z^{'},c \right)}{B(z,c)}$ (Eq 4).

$P_{repr}$ defines the ‘lifetime’ of an individual until it reproduces for the first time. The fundamental operator $N_{repr}$ of the modified reproduction-growth Kernel is then given by

$N_{repr}={(I- P_{repr})}^{-1}$ (Eq 5),

where $I$ is an identity matrix with the same dimensions as $P_{repr}$. The dominant eigenvalue of the fundamental operator $N_{repr}$ gives the mean number of censuses at which individuals appear before they have reproduced:

$ā_{repr}=e(N_{repr})-1$ (Eq 6)

The survival probability of a seedling until the age of first reproduction $ā_{repr}$ is then given by

$l_{\left( ā_{repr} \right)}\left( z_{0}, c \right)=(\int_{L}^{U} S\left( z_{0},c \right)\left( 1-B\left( z_{0} \right) \right)G\left( z^{'},z_{0},c \right)dzdc+\int_{L}^{U} S\left( z_{0},c \right)B\left( z_{0} \right)R\left( z^{'},z_{0} \right)dzdc)^ā_{repr}$ (Eq 7)

where $z_{0}$ is the relative size distribution of a initial cohort of seedlings of *F. alnus*. For more information on Markov chains in population models, we kindly refer to ^4,5^.

Supplementary References

1. Albrecht, J. *et al.* Logging and forest edges reduce redundancy in plant-frugivore networks in an old-growth European forest. *J. Ecol.* **101**, 990–999 (2013).

2. Schlautmann, J. *et al.* Observing frugivores or collecting scats: a method comparison to construct quantitative seed dispersal networks. *Oikos* **130**, 1359–1369 (2021).

3. Metcalf, C. J. E., Horvitz, C. C., Tuljapurkar, S. & Clark, D. A. A time to grow and a time to die: a new way to analyze the dynamics of size, light, age, and death of tropical trees. *Ecology* **90**, 2766–2778 (2009).

4. Ellner, S. P., Childs, D. Z. & Rees, M. *Data-driven Modelling of Structured Populations*. (Springer International Publishing, 2016). doi:10.1007/978-3-319-28893-2.

5. Caswell, H. *Matrix popultion models: construction, analysis, and interpretation*. (Sinauer Associates, 2001).
