# Supplementary material for: Common seed dispersers contribute most to the persistence of a fleshy-fruited tree
Source: Commun Biol. 2023 Mar 27;6:330. doi: 10.1038/s42003-023-04647-y (PMC10043030; doi:10.1038/s42003-023-04647-y)
Supplement: Supplementary file 3 — Reporting Summary [file 42003_2023_4647_MOESM3_ESM.pdf]

nature portfolio

Corresponding author(s): Rehling, Finn (COMMSBIO-22-2272)

Last updated by author(s): Sep 2, 2023

Reporting Summary

Nature Portfolio wishes to improve the reproducibility of the work that we publish. This form provides structure for consistency and transparency in reporting. For further information on Nature Portfolio policies, see our Editorial Policies and the Editorial Policy Checklist.

Statistics

For all statistical analyses, confirm that the following items are present in the figure legend, table legend, main text, or Methods section.

n/a	Confirmed	
☐	☒	The exact sample size (*n*) for each experimental group/condition, given as a discrete number and unit of measurement
☐	☒	A statement on whether measurements were taken from distinct samples or whether the same sample was measured repeatedly
☐	☒	The statistical test(s) used AND whether they are one- or two-sided<br>*Only common tests should be described solely by name; describe more complex techniques in the Methods section.*
☐	☒	A description of all covariates tested
☐	☒	A description of any assumptions or corrections, such as tests of normality and adjustment for multiple comparisons
☐	☒	A full description of the statistical parameters including central tendency (e.g. means) or other basic estimates (e.g. regression coefficient) AND variation (e.g. standard deviation) or associated estimates of uncertainty (e.g. confidence intervals)
☐	☒	For null hypothesis testing, the test statistic (e.g. F, t, r) with confidence intervals, effect sizes, degrees of freedom and P value noted<br>*Give P values as exact values whenever suitable.*
☒	☐	For Bayesian analysis, information on the choice of priors and Markov chain Monte Carlo settings
☒	☐	For hierarchical and complex designs, identification of the appropriate level for tests and full reporting of outcomes
☐	☒	Estimates of effect sizes (e.g. Cohen's *d*, Pearson's *r*), indicating how they were calculated

Our web collection on statistics for biologists contains articles on many of the points above.

Software and code

Policy information about availability of computer code

Data collection	R program version 4.1.1, data available here: https://doi.org/10.5061/dryad.h44j0zpmq.
Data analysis	R program version 4.1.1, code available here: https://doi.org/10.5061/dryad.h44j0zpmq.

For manuscripts utilizing custom algorithms or software that are central to the research but not yet described in published literature, software must be made available to editors and reviewers. We strongly encourage code deposition in a community repository (e.g. GitHub). See the Nature Portfolio guidelines for submitting code & software for further information.

Data

Policy information about availability of data

All manuscripts must include a data availability statement. This statement should provide the following information, where applicable:

- Accession codes, unique identifiers, or web links for publicly available datasets
- A description of any restrictions on data availability
- For clinical datasets or third party data, please ensure that the statement adheres to our policy

The original contributions and R code presented in the study will be made available online in the Dryad Digital Repository https://doi.org/10.5061/dryad.h44j0zpmq.

Human research participants

Policy information about studies involving human research participants and Sex and Gender in Research.

Reporting on sex and gender	This information has not been collected.
Population characteristics	See above.
Recruitment	See above.
Ethics oversight	See above.

Note that full information on the approval of the study protocol must also be provided in the manuscript.

Field-specific reporting

Please select the one below that is the best fit for your research. If you are not sure, read the appropriate sections before making your selection.

☐ Life sciences ☐ Behavioural & social sciences ☒ Ecological, evolutionary & environmental sciences

For a reference copy of the document with all sections, see nature.com/documents/nr-reporting-summary-flat.pdf

Ecological, evolutionary & environmental sciences study design

All studies must disclose on these points even when the disclosure is negative.

Study description

Quantifying long-term outcomes of mutualisms is difficult in real-world ecosystems due to its strong context-dependency, limiting our understanding of the functional role of species for the maintenance and conservation of biodiversity and ecosystems. In this study, we explored long-term effects of the seed dispersal mutualism between 20 frugivore species (14 birds and 6 mammals) and the population of the mid-successional tree Frangula alnus (glossy buckthorn) in the primeval Białowieża Forest, Poland.
We answered the following questions: (i) Do populations of animal-dispersed plants need animal dispersal to persist? (ii) What are the key factors determining the effectiveness of animal dispersers? (iii) Can the interactions of extinct species be functionally compensated by the remaining animal community

Short method description: We used integral projection models (IPMs) to study the effect of animal seed dispersal on the full life cycle of F. alnus along the gradient of canopy cover in the forest (Fig. 1, Supplementary Figure 1). The IPMs were partly built on datasets of previous studies: in a first step, we observed the removal of fruits, and the fruit handling behavior (i.e., removing or dropping fruits, and crushing seeds) by animal species over 936 h on 52 reproductive individuals of F. alnus (Fig. 1b) 51,52. In a second step, we determined animal-specific deposition patterns of seeds by collecting and DNA barcoding 1,729 scat samples with 9,590 seeds of F. alnus and other fleshy-fruited plants along the forest canopy gradient (Fig. 1c) 15,53. In a third step, we included an estimate of seed germination after gut passage from a recent meta-analysis in the models 11. In a fourth step, we investigated seedling recruitment by experimentally sowing 2,500 seeds 15. These comprehensive datasets were extended in this study by investigating the growth, survival and reproduction of 938 individuals of F. alnus along the forest canopy gradient over three years (Fig. 1d).

[1] Albrecht, J. et al. Logging and forest edges reduce redundancy in plant-frugivore networks in an old-growth European forest. J. Ecol. 101, 990–999 (2013).
[2] Schlautmann, J. et al. Observing frugivores or collecting scats: a method comparison to construct quantitative seed dispersal networks. Oikos 130, 1359–1369 (2021).
[3] Rehling, F., Schlautmann, J., Jaroszewicz, B., Schabo, D. G. & Farwig, N. Forest degradation limits the complementarity and quality of animal seed dispersal. Proc. R. Soc. B Biol. Sci. 289, 20220391 (2022).
[4] Rogers, H. S. et al. Frugivore gut passage increases seed germination: an updated meta-analysis. bioRxiv (2021) doi:10.1101/2021.10.12.462022.

Research sample

Study species: Frangula alnus Miller (Rhamnaceae) is distributed from Morocco throughout most of Europe to western Asia. It grows as a shrub or as small tree in open environments or in the understorey of mid-successional forests. In late-successional forests, shade-tolerant plants outcompete F. alnus, but its growth and regeneration may continue in canopy gaps. In Białowieża Forest, F. alnus produces fruits from the end of July to October. The black fruits have a diameter of 6.5 - 10.7 mm and contain on average two seeds with a mass of 21.2 mg (range 10.3-36.0 mg). Seeds are primarily dispersed by small birds and mammals, and secondarily by ants or water. They are physiologically dormant and both light and cold-stratification improve germination. Frangula alnus can produce clonal side-trunks and has the ability to resprout after breakage. Frangula alnus is one of the most abundant, fleshy-fruited plant species in Białowieża Forest. Frangula alnus was further the species, for which we had the most comprehensive set of replicates across all studied plant life stages of the 15 fleshy-fruited species of the plant community.

Sampling strategy

We recorded survival, growth and reproduction of practically all individiuals of F. alnus that were available at the study sites. When multiple plants occurred at the same location and were of similar size, we only investigated a random subset of the plant individuals.

Data collection

Seed removal observations
To quantify the interaction frequency of animal species with F. alnus, trained field staff (17 observers) recorded the seed removal and

fruit handling by frugivores at 15 sites during the fruiting period in 2011 and 2012. Frugivores visiting these individuals were observed with binoculars from camouflaged tents on three separate days for a period of 6 h starting at sunrise, i.e. for in total 936 h of observation. For each frugivore species, the number of visits, the number of fruits eaten during each of these visits and details of their fruit-handling were recorded. We differentiated between three types of fruit-handling: (i) swallowing or removing and (ii) dropping of fruits, and (iii) crushing of seeds. If groups of frugivores were visiting a tree at the same time, we recorded the number of visits and focused only on the behavior of one individual. Overall, 1,006 frugivore visits were observed and whether frugivores handled fruits or not was successfully observed in 766 of 821 cases (93.3%). JA coordinated the seed removal observations of F. alnus by animals.

Scat collection
To quantify seed deposition patterns by the animal species along the canopy gradient in the forest, we collected scats of frugivores containing seeds of any species of the associated fleshy-fruited plant community at 12 study sites from 2016 – 2018. At each study site, we set up five 100 m transects which were at least 20 m apart. Along each transect, we searched for animal scats within 1 m wide strips to the left and right of each transect, covering a total area of 1,000 m² per study site. The transects were checked every ten days during the fruiting season of the plant community lasting from mid-June to mid-October. After heavy rains, scat collection was paused for two days. At each study site, we collected scats eleven times in 2016 and 2018 and nine times in 2017 due to a shorter fruiting season. We collected all bird scats with seeds of the entire fleshy-fruited plant community to identify the frugivore in the laboratory. Mammal scats were assigned visually to species in the field. Seeds from mammal scats were counted in the field or also collected for genetic identification. The seeds from the scat samples were stored at -20 °C on the same day until they were used for frugivore identification in the laboratory. FR, JS and HF collected the scats.
To identify the frugivore species that had deposited the scat, we followed the DNA barcoding protocol of [1]. DNA extraction and the PCR amplification took place in the Conservation Ecology laboratory of the University of Marburg (Germany). DNA samples were then sent to LGC Genomics (Berlin, Germany) or Macrogen Europe (Amsterdam, Netherlands) for DNA purification and sequencing. The final DNA sequences were edited with CodonCode Aligner (Version 9.0.1, CodonCode Corporation) and species were identified using the Barcode of Life identification system (BOLD). For the analysis we only used samples whose sequences had a > 98 % similarity with recorded sequences in BOLD. We successfully identified the frugivore species for c. 90 % of our samples. JS conducted the DNA barcoding. We analyzed 1,729 scat samples and differentiated the seed deposition pattern of six animal species and one group of species collectively representing rare dispersers ('other'). Each of the deposition pattern was based on a minimum number of 30 scat samples.

Seedling recruitment
To assess the probability of a seed of F. alnus to develop into a seedling, we conducted recruitment experiments each year from 2016-2018. We collected fruits of at least six adults, removed the pulp, dried the seeds for 48 h at room temperature, and mixed them. At each of four study sites, we established 10 marked plots, and at each of these plots we established subplots for the different years of the recruitment experiments, 2016 (n = 40 plots), 2017 (n = 20, only half the plots) and 2018 (n = 40). We sowed 25 seeds per subplot, thus, 2,500 seeds in total. Each subplot had an area of 50×50 cm² and was at least 5 m away from the nearest reproductive F. alnus individual. From 2017 to 2019, we checked the experimental subplots for the number and size of seedlings once per year in June and then tracked their fate as part of the demographic study. In addition, we checked once for emerging seedlings of F. alnus on a control subplot next to each subplot where we had not sown seeds. We found only one seedling of F. alnus in these control plots indicating only negligible external seed input in our recruitment experiments. FR, JS, HF, LW conducted the sowing experiments and tracked the fate of seedlings.

Plant vital rates
To analyze the demography of F. alnus, we recorded survival, growth and reproduction of plant individuals during June-October from 2017-2019 at 14 study sites in the Białowieża Forest. To be able to locate plant individuals in the forest throughout the study period, we attached tags to randomly chosen individuals of all sizes at the study plots. We measured the stem diameter of tagged individuals of F. alnus at ground level with calipers or with a tape measure. We counted the number of fruits of adult trees before the main period of fruiting. However, in c. 27% of cases, we assessed a plant later in the season. Because animals had eaten some or even all of the fruits by that time, we only checked for leftovers of fruits to see if an individual was reproductive or not. We measured the diameter of only 65 of 253 first-year seedlings (26%). A large proportion of the plant individuals were not relocated in each census year because often tags were destroyed (c. 10-20%). Of the lost individuals, we were able to retrieve about 10 % of individuals in following study years. As a consequence, we recorded survival and death only for individuals which could be clearly identified throughout the study period. In total, we were able to record vital rates for 938 individuals, of which 341 were assessed once, 247 twice and 350 for three consecutive years (following outlier detection, see statistical analyses). FR, JS, HF and LW collected the demography data.

Canopy cover
For studying effects of canopy cover on seed deposition, we split each of the 100 m transects into five 20 m segments. Every scat that was found along these transects was assigned to the closest segment. We took up to six hemispherical photos of the canopy with a fisheye lens at ground level along the transects in 2016 and 2017. To study effects of canopy cover on plant demography, we tagged most plants close to the transects (within 10 m distance), and assigned these individuals to the closest segment of the transects. If plant individuals were located farther away from the transects, we took up to three hemispherical photos within 10 m of those plants from 2016 to 2019. At the same times, we also took photos at the center of each of the 40 plots used to study plant recruitment. All photos were taken during the fruiting period of the fleshy-fruited plant community from June-October. The hemispherical photos were analyzed with DHPT 1.0 to calculate the proportion of area that was covered by the canopy at each location. We used local averages of the canopy cover (across seasons and years) as explanatory variables in the analyses of the seed deposition patterns and the vital rates. JS took the hemispherical photos.

[1] González-Varo, J. P., Carvalho, C. S., Arroyo, J. M. & Jordano, P. Unravelling seed dispersal through fragmented landscapes: Frugivore species operate unevenly as mobile links. Mol. Ecol. 26, 4309–4321 (2017).

Timing and spatial scale

Timing: We leverage data from different years of field work in the same ecosystem: seed removal (2011-2012), seed deposition (2016-2018), plant recruitment (2017-2019), demography (2017-2019). In each year, we worked in the field from May until October, but in 2019 where the field measurements were not as comprehensive as in the other years (May-July).

Spatial scale: Our study took place in the Białowieża Forest at the border of Poland and Belarus, where the ash-alder floodplain forests (Fraxino-Alnetum community) are home to a diverse community of at least fifteen woody, fleshy-fruited plant species and at least 41 frugivorous animal species. All sampling took place at 17 study sites in ash-alder floodplain forests in both the managed (stand age: c. 70 years, n = 11) and the old-growth part (stand age: c. 100-150 years, n = 5) of the Białowieża Forest. Due to logistic constraints, we assessed each process important for the demography of F. alnus only at subsets of all study sites (Fig. 5, Supplementary Table 1-2): seed removal (at 15 sites), seed deposition (12 sites), seedling recruitment (4 sites, of which each site consisted of 10 plots, each with three subplots) and plant demography (14 sites). Our sites were distributed over an area of c. 400 km², i.e. two thirds of the Polish part of the Białowieża Forest.

Data exclusions

Seed deposition
Over the course of three years we found > 500 scats with seeds of Sambucus nigra beneath only one parental tree of S. nigra, as this tree produced over 40,000 fruits each year and attracted many frugivores. As seeds deposited beneath the S. nigra tree affected the overall pattern of seed deposition of frugivores when we pooled the deposition data, we used only scats with seeds of F. alnus from this transect segment in the analyses.

Vital rates
A large proportion of the plant individuals were not relocated in each census year because often tags were destroyed (c. 10-20%). Of the lost individuals, we were able to retrieve about 10 % of individuals in following study years. Often, thus, two or more different individuals had been incorrectly classified to be the same individual in the field, resulting in abnormal growth transitions (e.g. increases or decreases in diameter of > 10 cm). To reduce the probability of including false data, we identified potential outliers using 2.24*standard deviation of the studentized residuals of the global model of plant growth as a threshold. We removed 55 of the 1002 transitions of plant individuals from the dataset (c. 5.4% of total transitions): either entirely, if they had clearly erroneous values (n = 14), or by splitting up records of single individuals into that of two or more independent individuals (n = 41).

Reproducibility

Data and R code available in the Dryad Digital Repository.

Randomization

We collected 3,632 scats with 15,382 seeds (during the 2016-2018 seasons) of the plant-frugivore community, of which 375 scats contained seeds of F. alnus. We collected the most scats with seeds of F. alnus from the birds S. atricapilla (n = 232), T. merula (n = 55) and T. philomelos (n = 58), and another 30 scats from the remaining animal community. The low sample size of scats with seeds of F. alnus did not allow us to predict pair-wise seed deposition patterns of F. alnus for 17 of the 20 dispersers. To be able to include these animal species in the analyses, we made the following assumptions: (1) we pooled data on seed deposition of all animals across study sites and years in the forest assuming no temporal or spatial differences in our population. (2) We did not differentiate between seeds deposited beneath conspecific adults and elsewhere as early recruitment of seedlings was not affected by conspecific adults. (3) We assumed that frugivores showed the same behavior and dropped scats at the same locations along the canopy gradient independent of whether they had eaten fruits of F. alnus or one of the other 15 co-fruiting (i.e. temporally) plant species. Thus, scats with seeds of other species were considered equally representative for deposition patterns of the different frugivores across the canopy gradient, and could be used to study seed deposition pattern of F. alnus. We only pooled scats with seeds that were found at the same time as scats with seeds of F. alnus in each year (but for M. martes, see next point). (4) We found scats of M. martes at the same microhabitats (mostly the same tree logs) throughout the study years. This finding may be explained by the behavior of M. martes to mark home ranges using scats to communicate with other individuals. In order to have a sufficiently large sample size to analyze the deposition of seeds by M. martes, we pooled the scats with seeds of fleshy-fruited plants from the entire fruiting period. (5) In our IPMs, we analyzed the role of rare frugivores separately, but treated them as functionally equal to each other. We pooled all data on fruit removal and seed deposition for frugivores with < 10 scats. However, the number of replicates was still too low to analyze seed deposition by rare frugivores (n = 20). To inform the analysis, we included scats of animals that did not directly interact with F. alnus, but deposited seeds of co-occurring fleshy-fruited plants at the same period and location of the seed deposition of F. alnus. We assumed that the seed deposition by these animals, that are potentially able to interact with F. alnus, is equal to that of rare dispersers of F. alnus (n = 16 scats by six animal species). Only by analyzing the seed deposition by rare dispersers at the plant-frugivore community level, we were able to keep the analysis of contribution of these rare dispersers independent from frugivores that frequently interacted with F. alnus. We assume that these assumptions will not qualitatively affect the results of the study, as differences in seed deposition by frugivores were not very pronounced. In addition, small differences in seed deposition by frugivore species did not affect seed dispersal quality as strong as other components, for instance, seed predation.

We used bootstrapping to examine the uncertainty in the different demographic measures. We differentiated between uncertainty arising from observations of different plant individuals and uncertainty arising from differences in the dispersal process . To model uncertainty of dispersal, we resampled 500 times the removal observations, seed deposition and the seedling recruitment rates with replacement. To model uncertainty of growth, we resampled observations of plant individuals of F. alnus at each site in each year from the dataset, with replacement. By doing so, we created 500 datasets with three years of observations. The vital rates were analysed using the same model structure. The bootstrapped data had the similar structure as the original field data with respect to the number of replicates for each combination of year and site. In addition, we made sure that every disperser species was present in each bootstrap. From these, we calculated the frugivore-specific dispersal rates as described above. The structure of the formulas of the IPM were held constant for the vital rates, i.e. we only allowed parameter estimation for the most parsimonious model across bootstraps.

Blinding

Blinding was not relevant to our methods were standardized at each site following sampling protocols.

Did the study involve field work? ☒ Yes ☐ No

Field work, collection and transport

Field conditions

Białowieża Forest lies in the realm of a warm-summer humid continental climate. Mean annual precipitation for the period 1950–

Field conditions	2003 was 627.5 mm, with extreme values of 434.5 mm in 1964 and 898.0 mm in 1974. Most of the precipitation (391.1 mm) comes during the May–October. The mean snow cover period lasted for close to 80 days in 1950s, but declined to just 40 days in the last decade. The mean annual temperature (MAT) for the same period was 6.8 °C, with extreme values in 1996 (5.0 °C) and 1989 (8.9 °C). During the period 1950–2015 the MAT increased by 1.27 °C, with winter half-year warming much faster than the summer.
Location	Białowieża Forest is an approximately 1500 km² large forest complex stretching over the border between Poland and Belarus, in the eastern part of Central European Lowland (52°41′ N; 23°49′ E). It covers a flat plain with numerous shallow river valleys and a system of depressions and hills. The altitude ranges from 134 m–140 m, a.s.l. to 202 m a.s.l. The plain is a part of the larger moraine upland developed during retreat of the ice-sheet of the Warta Glaciation. The last glacial (Vistulian/Weichselian) ice-sheet did not cover the region, but periglacial processes resulted in covering older geological formations with younger sediments.<br><br>At present, the 630 km² of forest in Poland are divided into the Białowieża National Park (c. 105 km²) and forests managed by state forestry. In an area of about c. 48 km² of the Białowieża National Park, human interference has been minimal for over half a millennium and that part has been strictly protected since 1921, making it the best-preserved lowland forest in Europe. In contrast, since the First World War, commercial logging has shaped more than 80% of the Polish part of the forest that is not the national park. All sampling took place at 17 study sites in ash-alder floodplain forests in both the managed (stand age: c. 70 years, n = 11) and the old-growth part (stand age: c. 100-150 years, n = 5) of the Białowieża Forest. For further details on the exact locations of the 17 study sites, see Figure 5.
Access & import/export	Before we accessed the Białowieża Forest each year, we were in close contact with the administration of the Białowieża National Park, the forest administrations of Białowieża, Hajnówka and Browsk, and Polish authorities (Ministry of Environment, GDOS [Polish General Directorate of Environment Conservation, Warsaw] and RDOS [Regional Directorate of Environment Conservation, Białystok]) for the permission to research. BJ (Białowieża Geobotanical Station) organized and administrated the field work in Białowieża Forest throughout the study period. In yearly reports, we informed the authorities about the current status of research, the number and identity of study samples, and the future plans of the project.<br><br>We collected data in the Białowieża National Park under the research permit ZE.5000.02.2017 and in the state forests under the research permit WPN.6205.20.2017.MN; "Wpływ użytkowania ekosysteómow lesnych na procesy ekosystemowe i mutualistyczne sieci zależnosci w lasach mieszanych klimatu umiarkowanego Wschodniej Polski". Reports and permissions were send to authorities in January-March each year.
Disturbance	During the study period, we only entered the forest when we recorded data and in coordination with the local authorities. We only used indirect methods to study the seed removal and deposition of animals. For the sowing experiment, not more fruits were collected than needed for the study design. In addition, only seeds from the old-growth part of the forest were used for the recruiment experiments in the old-growth forest. For the tree inventory, we measured plants without injuring the tree. We always used the same tracks to go through the forest matrix. After the study period ended, we removed all tags from the plants. By doing so, we kept the disturbance within the forest to a minimum.

Reporting for specific materials, systems and methods

We require information from authors about some types of materials, experimental systems and methods used in many studies. Here, indicate whether each material, system or method listed is relevant to your study. If you are not sure if a list item applies to your research, read the appropriate section before selecting a response.

Materials & experimental systems

n/a	Involved in the study	
☒	☐	Antibodies
☒	☐	Eukaryotic cell lines
☒	☐	Palaeontology and archaeology
☐	☒	Animals and other organisms
☒	☐	Clinical data
☒	☐	Dual use research of concern

Methods

n/a	Involved in the study	
☒	☐	ChIP-seq
☒	☐	Flow cytometry
☒	☐	MRI-based neuroimaging

Animals and other research organisms

Policy information about studies involving animals; ARRIVE guidelines recommended for reporting animal research, and Sex and Gender in Research

Laboratory animals	The study did not involve laboratory animals.
Wild animals	We observed indirectly 26 animal species removing fruits of Frangula alnus, depositing seeds of Frangula alnus, or depositing seeds of fleshy-fruited plants other than Frangula alnus during the same period.<br><br>These animal species were Apodemus flavicollis, Bison bonasus, Cervus elaphus, Coccothraustes coccothraustes, Columba palumbus, Dendrocopos major, Dendrocopos medius, Dryomys nitedula, Erithacus rubecula, Garrulus glandarius, Luscinia luscinia, Martes martes, Muscardinus avellanarius, Muscicapa striata, Myodes glareolus, Parus major, Prunella modularis, Pyrrhula pyrrhula, Sciurus vulgaris, Sitta europaea, Sus scrofa, Sylvia atricapilla, Sylvia borin, Turdus merula, Turdus philomelos, and an unknown Phasianidae.

Reporting on sex	This information has not been collected.
Field-collected samples	We collected all bird scats with seeds of the 15 fleshy-fruited plant species to identify the frugivore in the laboratory. Seeds from mammal scats were counted in the field or also collected for genetic identification. The seeds from the scat samples were stored at -20 °C in the dark on the same day until they were used for frugivore identification in the laboratory.
Ethics oversight	No ethical approval was required, as we either only studied animals indirectly (observations with a binocular, scat collections), or studied plants.

Note that full information on the approval of the study protocol must also be provided in the manuscript.
